# The effect of temperature on reproduction in the summer and winter annual *Arabidopsis thaliana* ecotypes Bur and Cvi

**DOI:** 10.1093/aob/mcu014

**Published:** 2014-02-25

**Authors:** Ziyue Huang, Steven Footitt, William E. Finch-Savage

**Affiliations:** School of Life Sciences, Wellesbourne Campus, University of Warwick, Warwickshire CV35 9EF, UK

**Keywords:** Maternal temperature, seed yield, seed dormancy, flower development, *Arabidopsis thaliana* ecotypes, winter annual, summer annual, Cvi, Burren, climate change

## Abstract

**Background and Aims:**

Seed yield and dormancy status are key components of species fitness that are influenced by the maternal environment, in particular temperature. Responses to environmental conditions can differ between ecotypes of the same species. Therefore, to investigate the effect of maternal environment on seed production, this study compared two contrasting *Arabidopsis thaliana* ecotypes, Cape Verdi Isle (Cvi) and Burren (Bur). Cvi is adapted to a hot dry climate and Bur to a cool damp climate, and they exhibit winter and summer annual phenotypes, respectively.

**Methods:**

Bur and Cvi plants were grown in reciprocal controlled environments that simulated their native environments. Reproductive development, seed production and subsequent germination behaviour were investigated. Measurements included: pollen viability, the development of floral structure, and germination at 10 and 25 °C in the light to determine dormancy status. Floral development was further investigated by applying gibberellins (GAs) to alter the pistil:stamen ratio.

**Key Results:**

Temperature during seed development determined seed dormancy status. In addition, seed yield was greatly reduced by higher temperature, especially in Bur (>90 %) compared with Cvi (approx. 50 %). The reproductive organs (i.e. stamens) of Bur plants were very sensitive to high temperature during early flowering. Viability of pollen was unaffected, but limited filament extension relative to that of the pistils resulted in failure to pollinate. Thus GA applied to flowers to enhance filament extension largely overcame the effect of high temperature on yield.

**Conclusions:**

High temperature in the maternal environment reduced dormancy and negatively affected the final seed yield of both ecotypes; however, the extent of these responses differed, demonstrating natural variation. Reduced seed yield in Bur resulted from altered floral development not reduced pollen viability. Future higher temperatures will impact on seed performance, but the consequences may differ significantly between ecotypes of the same species.

## INTRODUCTION

The two major transitions of the plant life cycle are germination (seed to plant transition) and reproduction (plant to seed transition). The timing of these events is under strong genetic and environmental control, with increasing evidence that the two events are linked (Chiang *et al.*, 2009; Wilczek *et al.*, 2009; Wigge, 2011; Kendall and Penfield, 2012). As our understanding of climate change and the potential impact it may have on plant life cycle characteristics develops, there is an enhanced need to understand the impact on the reproductive phase (Hedhly *et al.*, 2009; Zinn *et al.*, 2010). The resulting seed traits are key components of plant fitness that are determined by their genotype and the maternal environment (Luzuriaga *et al.*, 2006).

Several studies have shown that maternal temperature is the major factor influencing seed production and subsequent germination behaviour (Hedhly *et al.*, 2003; Rutter and Fenster, 2007; Hedhly *et al.*, 2009), and a range of seed responses in different species have been summarized by Baskin and Baskin (1998) and Fenner (1991). For example, temperature can affect seed size in some species such as *Plantago lanceolata* (Alexander and Wulff, 1985), *Desmodium paniculatum* (Wulff, 1986) and *Chenopodium rubrum* (Cook, 1975). Maturation temperature also influences seed dormancy and germination, but the effects differ between species. Higher temperatures during seed development result in decreased seed dormancy in *Aegilops ovate* (Datta *et al.*, 1972), *Arabidopsis thaliana* (Kendall *et al.*, 2011; Kendall and Penfield, 2012), *Avena fatua* (Sawhney *et al.*, 1985) and *Glycine max* (Keigley and Mullen, 1986), whereas the reverse occurred in other species such as *Syringa reflex* and *Themeda australis* (Junttila, 1973; Groves *et al.*, 1982). It is also increasingly apparent that not only is the environment directly experienced by the developing seed important, but the parent plant is able to transmit signals that provide the seed with environmental information (Fenner, 1991; Kendall and Penfield, 2012).

Plant development, particularly the reproductive processes such as pollen development, pollen tube growth and fruit set, is highly vulnerable to environmental conditions, particularly temperature. These effects have potentially serious impacts on crop yield and can determine the potential to drive adaptation of reproductive traits to compensate for future temperature increases (Hedhly *et al.*, 2009; Zinn *et al.*, 2010). For example, the negative impact of higher temperatures on reproduction can lead to reduced pollen production, viability and pollen tube growth, with a resulting decrease in seed yield (Zinn *et al.*, 2010). High temperature can also induce flower abortion in arabidopsis and *Brassica juncea* (Gan *et al.*, 2004; Warner and Erwin, 2005).

Arabidopsis ecotypes exhibit adaptive differentiation in response to the environment (Rutter and Fenster, 2007). Such developmental responses to seasonal variation in the environment are important in understanding how plants adapt their life histories in nature (Donohue, 2009). In this study, the effects of maternal environment, particularly temperature, were studied using two arabidopsis ecotypes, Burren (Bur) and Cape Verdi Island (Cvi), that are adapted to very different ‘home’ environments and therefore exhibit summer and winter annual phenotypes, respectively. Bur is adapted to a cool and wet climate (Evans and Ratcliffe, 1972); in contrast, Cvi is adapted to a warm and dry climate (Evans and Ratcliffe, 1972; Footitt *et al.*, 2013). The application of contrasting environmental conditions rather than a single ‘control’ environment facilitates the study of ecotype plasticity (Sultan, 2004). We therefore grew Bur and Cvi plants in reciprocal controlled environments that simulated their native environments to investigate the influence of maternal temperature on reproductive development, seed production and subsequent germination behaviour.

## MATERIALS AND METHODS

### Plant material and growth conditions

Growth medium (Levingtons F1 compost:sand:vermiculite 6:1:1) was added to P24 cellular trays (24 cells, each 5 × 5 × 5 cm) placed in a second tray lined with capillary matting to ensure all the plants had a uniform water supply from below. Approximately five seeds were sown in each cell. The trays were then placed in a growth cabinet and covered with transparent propagator lids for at least 4 d, by which time all the seedlings had established. One week after sowing, the seedlings were thinned to one per cell. Eight trays of each of the *Arabidopsis thaliana* ecotypes, Bur and Cvi, were grown in a common environment (23/17 °C, 12/12 h, light/dark, 100 µm m^−2^ s^−1^ white light) until they bolted. Bolting was defined as the primary inflorescence reaching 1 cm long. Following bolting, four trays of each ecotype were placed in each of two environmental regimes that represented the daily temperature regime and daylength characteristics of the Burren region of Ireland and the Cape Verde Islands during flower and seed development: Bur environment (17/10 °C, 14/10 h, light/dark) and Cvi environment (27/22 °C, 12/12 h, light/dark). When the majority of siliques had turned yellow, the plants were no longer watered and 7 d later mature seeds were harvested.

### Seed harvesting and yield measurement

In all cases, seeds were harvested from individual plants as seeds became fully mature (i.e. when all the siliques had turned yellow and dry on the plant). Harvested seeds were equilibrated to 55 % relative humidity/20 °C for 6 d, resulting in a seed moisture content of 8–10 % on a dry weight basis, then seed yield (total seed weight) and seed size (1000-seed weight) were determined. Seeds were then sealed in air-tight tubes and stored at –80 °C.

### Dormancy and germination assays

Seeds were surface sterilized in a 0·125 % sodium hypochlorite solution [household bleach (5 % sodium hypochlorite) diluted to 2·5 % (Domestos^®^, Unilever)] for 5 min, washed three times with distilled water and then incubated on two layers of 3MM chromatography paper in rectangular clear plastic boxes (8 × 12 cm, Stewart Plastics) containing 8 mL of distilled water. For each treatment, there were three replicates of 40 seeds of each ecotype. Seeds where then incubated at 10 and 25 °C in the light for up to 28 d. Germination was recorded at regular intervals. A seed was considered to have germinated when the radicle had emerged through the testa and endosperm.

### Flower and silique development

A second cohort of Bur plants were grown in the common regime and then transferred at bolting to the respective native growth regimes: (1) Bur environment (Bur/Bur); (2) Cvi environment (Bur/Cvi); (3) flowers on plants in the Cvi environment were left untouched or sprayed three times with 10 µm gibberellin 4 + 7 (GA_4+7_) in 1·7 mm citric acid/3·3 mm K_2_HPO_4_ buffer at pH 5·0 at 2 d intervals (Bur/Cvi + GA); or (4) or were hand pollinated by pollen from Bur/Cvi plants (Bur/Cvi + Hand pollination). Trays containing Bur/Cvi + GA and Bur/Cvi + Hand pollination plants were isolated using perforated plastic bread bags to prevent cross-pollination. Each day flowering buds were marked by applying different coloured acrylic paint to the pedicel, and flowers collected at each developmental stage (stages 13–15) (Smyth *et al.*, 1990) were dissected. Flowering was defined as the first appearance of petals from within the enclosing sepals. Sepals and petals were removed to expose the anthers and pistils. The lengths of the pistil and stamen were measured to determine the pistil:stamen ratio (Footitt *et al.*, 2007). Siliques were dissected to determine the final seed set; the developed ovules present within each silique were counted to determine the percentage fertilization.

### Data analysis

Data are presented as the mean ± standard error, where standard error is the standard deviation divided by the square root of the number of replicates (*n*) (s.d./√*n*). Analysis of variance (ANOVA) was used to detect the differences between variates, including bolting time, leaf number at bolting, seed yield, 1000-seed weight, and length of pistils and stamens. All percentage germination data were angular transformed [arcsin(*x*)] for analysis. A two-sample unpaired *t*-test was used to show the significance of the results of silique length after flowering on each day, using the cut-off of *P* < 0·05 to determine statistical significance. Statistical analysis was carried out using the software package GenStat (VSN International, 2012).

## RESULTS

### Vegetative phenotypes of Bur and Cvi

**Fig. 1.**
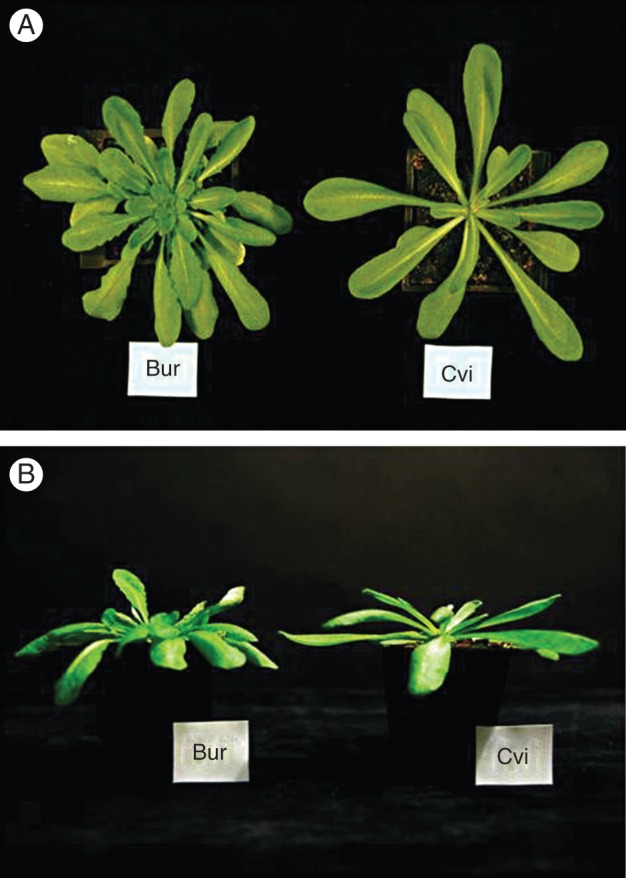
Plant phenotypes at 28 d after sowing in the common environment: 23/17 °C, 12/12 h, light/dark. (A) Overhead view of the Bur and Cvi rosettes; (B) side view of the Bur and Cvi rosettes.

**Fig. 2.**
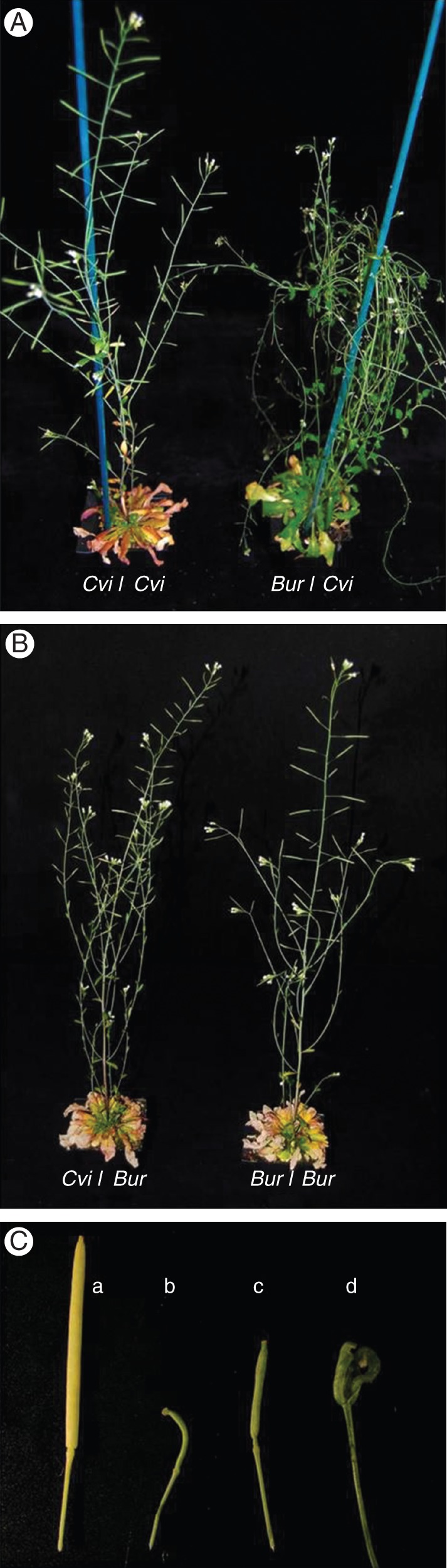
Plant phenotypes during reproductive growth. (A) Cvi and Bur plants grown in the Cvi environment; (B) Cvi and Bur plants grown in the Bur environment; (C) siliques of Bur plants developed in the Cvi environment (a, well-developed silique; b–d, curly and withered siliques).

Plants were grown in the same common environmental regime (23/17 °C, 12/12 h, light/dark) until bolting. There were clear physiological and morphological differences between the two ecotypes throughout growth and development. During the vegetative stage, the rosette leaves of Cvi plants were obovate with an entire margin, whereas the Bur leaves had a slightly serrated margin (Fig. 1A). The rosette leaves of Bur were less rigid than those of Cvi at this stage (Fig. 1B). Cvi plants had a mean bolting time (34·8 ± 0·2 d) that was significantly (*P* < 0·001) earlier than that of Bur plants (46·3 ± 0·3 d). Cvi plants also had fewer rosette leaves (18 0 ± 0·2) at bolting than Bur plants (64·5 ± 1·0). Both ecotypes reached a final height of 20–25 cm. During reproductive growth, Cvi plants grown in the Bur environment (Cvi/Bur) were not greatly different from those grown in their native environment (Cvi/Cvi; Fig. 2A, B). However, Bur plants in the Cvi environment (Bur/Cvi) had more branched inflorescences than Cvi (Fig. 2A) or Bur in its own environment (Bur/Bur; Fig. 2B). Bur/Cvi plants also had poorly developed siliques that were curled or withered, with few or no seeds (Fig. 2C).

### Seed yield

**Fig. 3.**
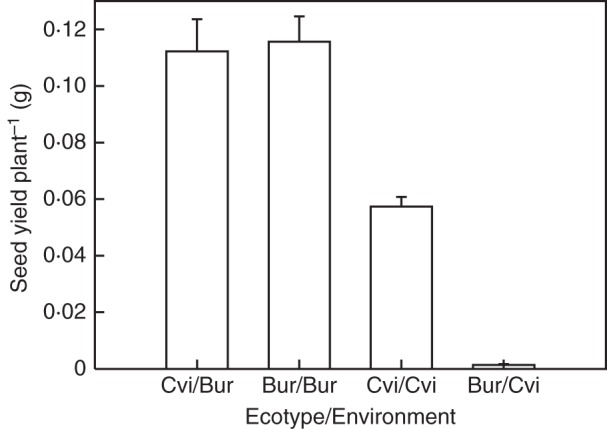
Seed yield per plant in simulated Bur and Cvi environments. Data are the mean ± s.e. (*n* = 24).

Both environment and ecotype had significant effects on final seed yield (*P* < 0·001 and *P* < 0·01, respectively). In the warmer Cvi environment, plants of both ecotypes yielded fewer seeds when compared with those in the cooler Bur environment. Seed yield of the Cvi ecotype in its native environment only reached half of the final yield achieved in the Bur environment (Fig. 3). Seed yield of the Bur ecotype was also significantly (*P* < 0·001) different in the two environments, being dramatically reduced under Cvi conditions. These Bur/Cvi plants also produced significantly fewer (*P* < 0·01) but larger seeds (i.e. higher 1000-seed weight; *P* < 0·001) than either Cvi or Bur grown in its native environment (Table 1). Cvi seed size was not significantly different between the two environments, but this ecotype produced significantly more seeds in the cooler Bur environment than in the Cvi environment. In contrast, both seed size and number for the Bur ecotype were significantly different (*P* < 0·01) in the two environments (Table 1).

**Table 1.** 1000-seed weight and number of seeds produced per plant

Ecotype/environment	1000-seed weight (mg ± s.e.)	Seed number (×10^3^ per plant ± s.e.)
Bur/Bur	30·1 ± 0·719	3·843 ± 0·289
Bur/Cvi	37·3 ± 1·767	0·039 ± 0·008
Cvi/Bur	32·6 ± 0·580	3·434 ± 0·327
Cvi/Cvi	29·7 ± 0·171	1·930 ± 0·112
l.s.d. (5 %)	3·535	0·5671

Data are means ± s.e.

The l.s.d. values are the least significant difference at *P* < 0·05.

### Seed germination responses to temperature

**Fig. 4.**
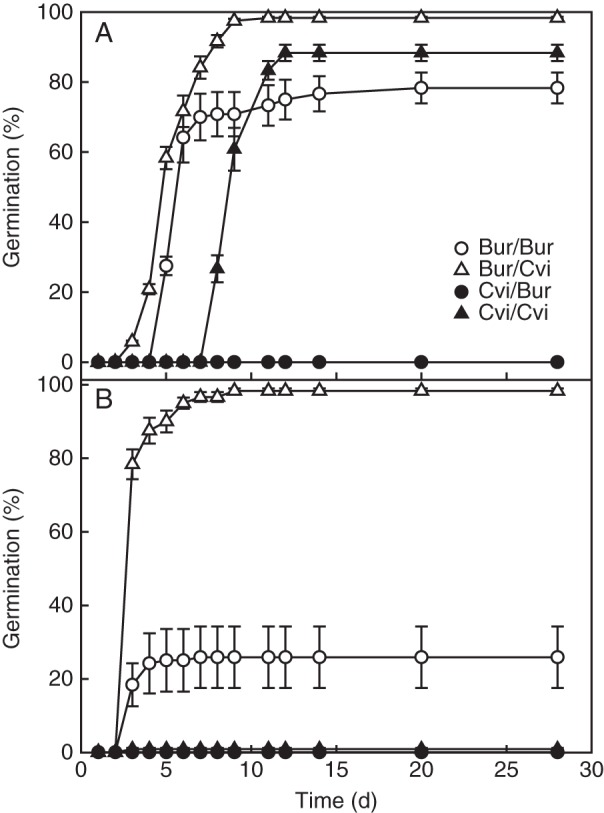
Seed germination responses at 10 °C (A) and 25 °C (B) of Bur and Cvi seeds produced in both Bur and Cvi environments. Data are the mean ± s.e. (*n* = 3). No error bar indicates that the symbol is larger than the error.

Thermo-dormancy and germination potential were characterized at 10 and 25 °C in the light. There was a significant (*P* < 0·001) effect of ecotype, production conditions and germination temperature. Although Bur/Cvi plants yielded few seeds, they had the highest germination percentage at both 10 and 25 °C (Fig. 4). In contrast, Cvi/Bur seeds did not germinate at either 10 or 25 °C. Bur/Bur seeds had a higher percentage germination at 10 °C than at 25 °C. Similarly, Cvi/Cvi seeds germinated to higher percentages at 10 °C than at 25 °C. The time to 50 % germination (*T*_50_) was calculated for seeds that reached >50 % final germination (Table 2). Bur/Cvi seeds had the lowest *T*_50_ (i.e. high germination rate), germinating slightly faster than Bur/Bur seeds (not significant). Cvi/Cvi seeds had the highest *T*_50_ (i.e. low germination rate).

**Table 2.** Final germination percentage and time in days to 50 % germination (*T*_50_) of arabidopsis seeds at 10 and 25 °C

Ecotype/environment	Final germination (% ± s.e.)		*T*_50_ (d)	
	10 °C	25 °C	10 °C	25 °C
Bur/Bur	78·3 ± 4·4	25·8 ± 8·35	7·3 ± 2·0	–*
Bur/Cvi	98·3 ± 0·33	98·3 ± 0·67	5·0 ± 0·3	2·8 ± 0·1
Cvi/Bur	0	0	–	–
Cvi/Cvi	88·3 ± 2·33	0·8 ± 0·33	8·8 ± 0·4	–

Data are the mean ± SE (*n* = 3).

*The missing data indicate that the germination did not reach 50 %, so there was no *T*_50_ value.

### Floral development in the Bur ecotype

**Fig. 5.**
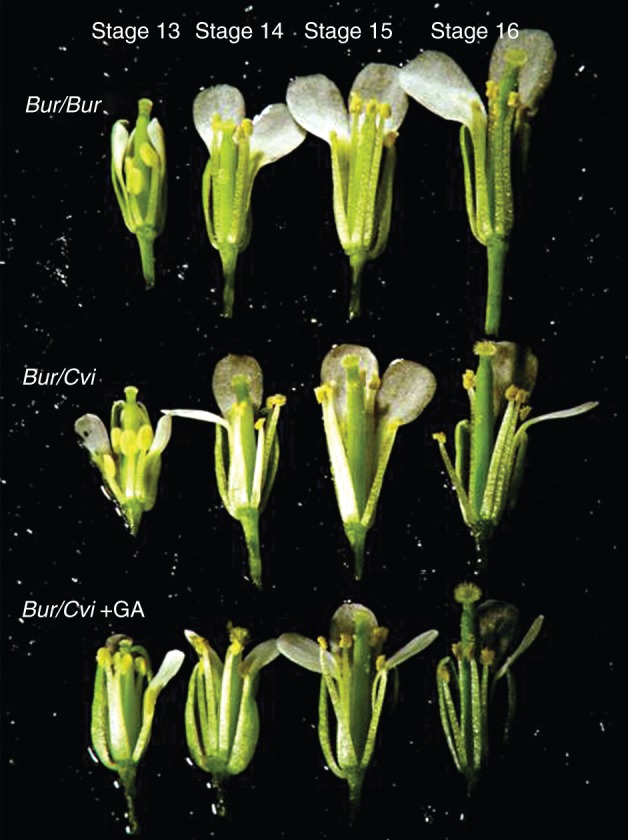
Partially dissected Bur flowers (stages 13–16) developed in the Bur (Bur/Bur) and Cvi environments (Bur/Cvi), and treated with a 10 µm GA_4+7_ solution in the Cvi environment (Bur/Cvi + GA).

**Fig. 6.**
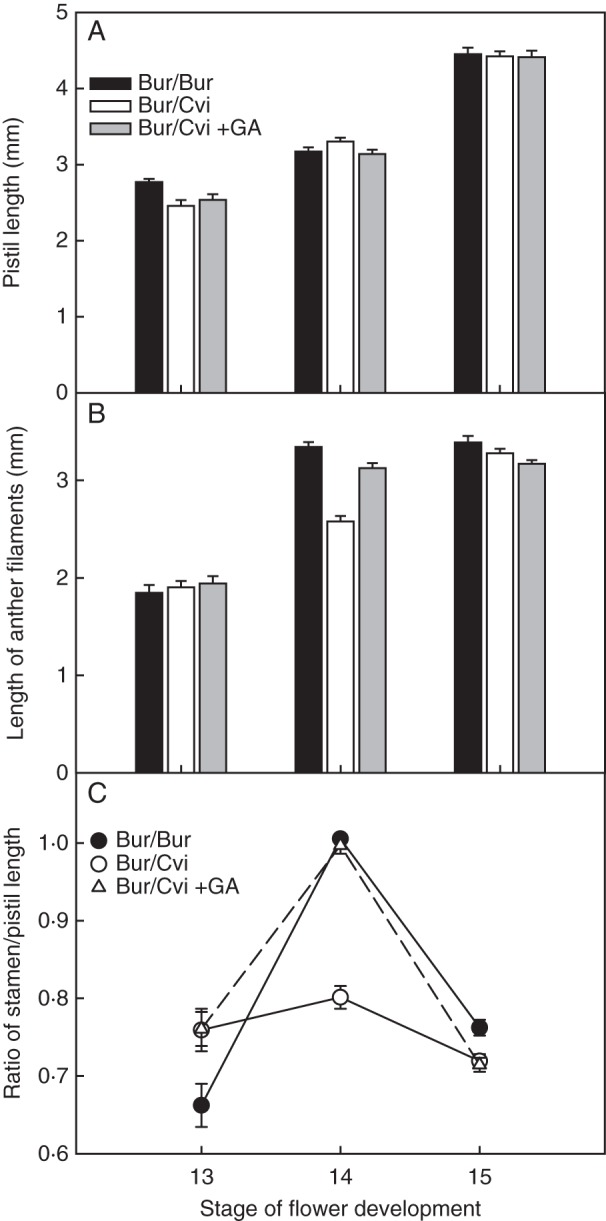
Floral phenotypes of the Bur ecotype at different developmental stages in both Bur and Cvi environments. (A) Pistil lengths; (B) stamen lengths; (C) stamen/pistil ratio. Data are the means ± s.e. (floral stage 13, *n* ≥19; floral stage 14, *n* ≥28; floral stage 15, *n* ≥20). No error bar indicates that the symbol is larger than the error.

Following the observation that seed yield was reduced in Bur/Cvi plants, flowers of Bur plants grown in both environments were dissected to see if flower morphology was altered. In both environments, morphology appeared normal, with the exception of stage 14 when the anthers typically extend above the stigma so that pollination can occur (Fig. 5). In Bur/Bur plants, stamens extend past the pistil to facilitate pollination. However, during this stage, Bur/Cvi plants had pistils longer than the stamens, even during the fertilization period. Therefore, flowers may have failed to be adequately pollinated because the length difference between pistil and stamens throughout growth limited the transfer of pollen (Fig. 5). When these Bur/Cvi flowers were sprayed with GA_4+7_ to stimulate the growth of stamens, there was adequate pollination during the fertilization stage (Fig. 5). The lengths of pistils and stamens were measured at stages 13, 14 and 15 (Smyth *et al.*, 1990) and the stamen/pistil length ratio was calculated. Pistil lengths were significantly (*P* > 0·001) different between stages regardless of the environment in which Bur plants were grown. During the fertilization period (stage 14), the mean pistil length of Bur/Cvi plants was longer than those in the Bur environment and those to which GA solution was applied (Fig. 6A), but the difference was not significant. However, the mean stamen length in Bur/Cvi plants was significantly shorter (*P* < 0·001) than that in the other two environments (Fig. 6B).

Both the stage of flower development and the growth environment had significant effects (*P* < 0·001) on the ratio of stamen to pistil length (Fig. 6C). The difference in the stamen/pistil length ratio was significant between the Bur and Cvi environment, but was not significant between the flowers grown in the Bur environment and those treated with GA in the Cvi environment. The ratio was >1·0 in flowers developing in the Bur environment and in flowers treated with GA, but that of flowers in the Cvi environment was always <1·0 (Fig. 6C). This result, combined with the data on stamen length (Fig. 6B) and hand pollination with Bur/Cvi pollen (Fig. 8), shows that the main cause of poor seed production was abnormal development of stamens in the higher temperature of the Cvi environment, resulting in reduced pollination.

### Silique development in the Bur ecotype

**Fig. 7.**
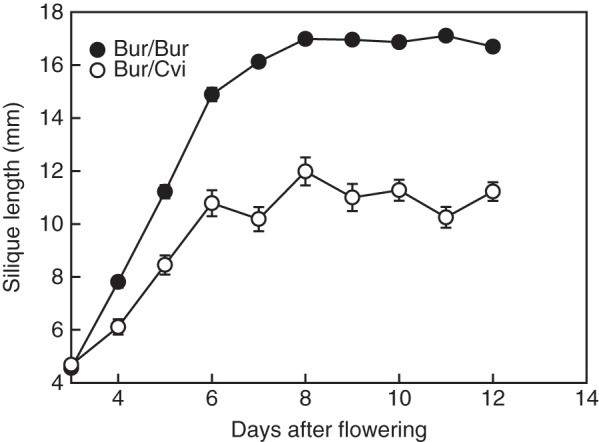
Silique length in Bur plants after flowering in both the Bur (*n* = 57) and Cvi (*n* = 56) environments. Data are the mean ± s.e. No error bar indicates that the symbol is larger than the error.

**Fig. 8.**
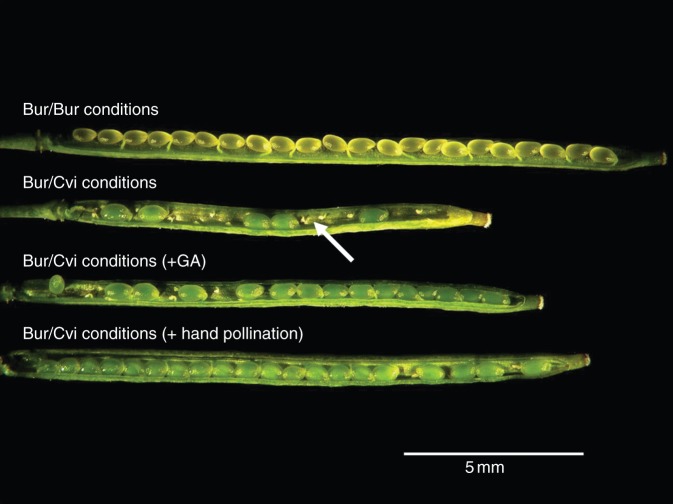
Partially dissected siliques of the Bur ecotype in different environments: Bur plants grown in both Bur and Cvi environments, and Bur/Cvi treated with 10 µM GA_4+7_ solution (+GA) or hand pollinated. The white arrowhead indicates an unfertilized ovule in a Bur/Cvi silique.

**Fig. 9.**
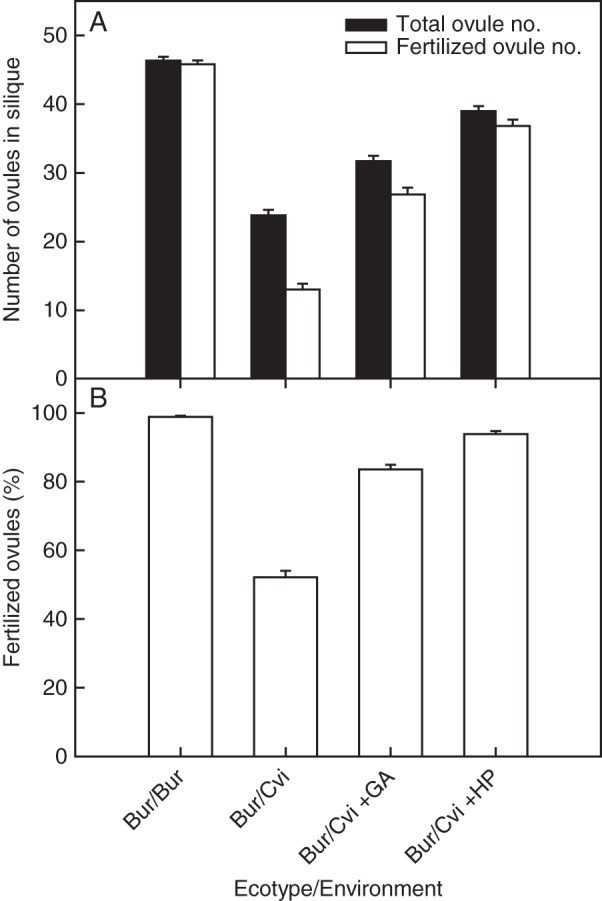
The impact of simulated Bur and Cvi environments on ovule development. (A) Total number of ovules and number of fertilized ovules; (B) percentage of ovules fertilized in siliques of Bur plants grown in both Bur (*n* = 57) and Cvi (*n* = 56) environments, and Bur/Cvi treated with 10 µM GA_4+7_ solution (+GA; *n* = 57),) or hand pollinated (HP; *n* = 68). Data are the mean ± s.e. No error bar indicates that the symbol is larger than the error.

Silique development was monitored after flowering. During the early stage (3 d after flowering), Bur silique length was similar in both environments. From 4 d after flowering, the lengths of Bur siliques developed in the Cvi environment were significantly shorter (*P* < 0·05), achieving only half the final length of siliques produced in the Bur environment (Fig. 7). The siliques of Bur/Bur and Bur/Cvi plants were dissected and this revealed that, unlike those from Bur/Bur plants, unfertilized ovules appeared to predominate toward the base of the Bur/Cvi siliques (shown with white arrowhead in Fig. 8). However, when siliques were treated with GA or hand pollinated, more ovules were fertilized. Both the total number of ovules and the percentage of developed ovules within each Bur silique were significantly (*P* < 0·001) affected by the growth environment and treatments, with ovule number significantly reduced in the Cvi environment (Fig. 9A). In Bur/Bur siliques, 99 % of ovules were fertilized, but in those of Bur/Cvi only 53 % of ovules were fertilized (Fig. 9B). When flowers were treated with GA or hand pollinated, both the total number of ovules and the number of fertilized ovules increased significantly in Bur/Cvi siliques (Fig. 9A), leading to a significant (*P* < 0·001) increase in the percentage of fertilized ovules to 84 and 94 %, respectively (Fig. 9B), confirming that Bur pollen was viable under these conditions.

## DISCUSSION

Natural variation between populations of the same species adapted to diverse seasonal environments can be used to investigate both the genetic and environmental link between these transitions and subsequent life cycle behaviour (Donohue *et al.*, 2005; Donohue, 2009). To this end, the increasing use of the natural variation in arabidopsis to identify variation and map adaptive traits has produced significant advances in our understanding (Koornneef *et al.*, 2004; Schmuths *et al.*, 2006; Alonso-Blanco *et al.*, 2009; Weigel, 2012). In this study we used the natural variation between arabidopsis ecotypes with summer annual (Bur) and winter annual (Cvi) behaviour that are adapted to cool and warm climates, respectively, to determine the temperature sensitivity of the reproductive phase.

### The impact of maternal temperature on seed yield and size is ecotype dependent

Plants of both ecotypes produced more seeds when the reproductive phase took place in the Bur environment (low temperature) than in the Cvi environment (high temperature), in agreement with work showing that seed yield decreased when soybean, canola and *Arachis hypogaea* plants were exposed to high temperature (Gibson and Mullen, 1996; Prasad *et al.*, 2003; Gan *et al.*, 2004). Maternal environment also had an impact on seed size, but the effect differed between ecotypes, with higher temperature leading to smaller seeds in Cvi and larger seeds in Bur. Seed size presents a fundamental trade-off between producing more small seeds vs. fewer large seeds from a given quantity of resource allocated to reproduction (Smith and Fretwell, 1974). Since higher temperature experienced by the mother plants significantly reduced the seed yield in Bur, the larger and better provisioned seeds produced probably represent a strategy to increase the chances of successful seedling establishment.

### Both the maternal environment and genotype determine seed dormancy in *Arabidopsis thaliana*

When seeds of the two ecotypes were produced in the same environment they had different depths of dormancy, consistent with a genetic cause. However, in both ecotypes, seeds produced in different environments also have different depths of dormancy, consistent with a maternal environment influence. Bur seeds produced in the cooler Bur environment have deeper dormancy than those produced in the warmer Cvi environment. Further, Cvi seeds produced in the Bur environment did not germinate at either temperature, whereas they germinated at 10 °C when produced in the warmer Cvi environment. Thus enhanced primary dormancy induced by lower production temperatures in the maternal environment may well be a conserved response. Thus, consistent with other reports (Donohue, 2009; Kendall and Penfield, 2012; Penfield and Springthorpe, 2012), combinations of both genotype and the environment during seed maturation determine depth of dormancy in arabidopsis. This has been related to the temperature sensitivity of *DOG1* (*DELAY OF GERMINATION1*) expression during seed maturation (Chiang *et al.*, 2011; Nakabayashi *et al.*, 2012). Maternal environment also influences the germination and dormancy of seeds in a number of other species (Baskin and Baskin, 1988); for example, increased germinability in response to higher maternal temperatures in Cotton thistle (*Onopordum acanthium*) (Qaderi *et al.*, 2003), and ryegrass (*Lolium rigidum*) (Steadman *et al.*, 2004).

### Decreased seed yield mainly results from reduced stamen extension at high temperatures

It is widely accepted that plant reproduction is highly sensitive to environmental factors such as temperature (Hedhly *et al.*, 2003, 2009; Zinn *et al.*, 2010). Here we investigated whether reduced seed yield was a consequence of the reduced pollen viability resulting from temperature stress that has been shown to reduce seed yield in a number of crops such as *A. hypogaea* (Prasad *et al.*, 2003), *Cicer arietinum* (Srinivasan *et al.*, 1998), cowpea (*Vigna unguiculata*) (Hall, 2004) and *Capsicum* species (Reddy and Kakani, 2007). We found that hand pollination was effective in increasing the percentage of fertilized Bur ovules in the higher temperature Cvi environment from 52·9 to 93·9 %, indicating that temperature did not compromise the pollen. However, higher temperature had a significant negative impact on stamen growth, but no concomitant impact on pistil growth. Thus lack of filament growth resulted in anthers not extending above the stigma by stage 14 of flower development. In consequence, limited deposition of pollen on to the receptive pistil reduced pollination.

Gibberellin is a general regulator of floral development, and GA-deficient mutants typically have short stamens as a result of reduced cell extension within the filament (Cheng *et al.*, 2004). The growth-repressing DELLA proteins RGA, RGL1 and RGL2 work together to repress stamen and anther development in GA-deficient plants (Cheng *et al.*, 2004). In rice susceptible to temperature-induced sterility, anthers had lower GA levels than those in a temperature-tolerant rice line (Tang *et al.*, 2008). Similarly the male-sterile arabidopsis mutant MS33 which displays inhibition of stamen filament growth had low GA levels (Fei *et al.*, 2004). High levels of expression of the GA biosynthesis gene GA 20-oxidase (*GA20ox*), especially of the *GA20ox1* paralogue, are seen in developing stamens. The Bur ecotype carries a loss-of-function mutation in the *GA20ox4* paralogue. However, in Col-0, expression of this paralogue does not increase in filaments of the *GA20ox1* × *Ga20ox2* double mutant (Plackett *et al.*, 2012). Whether the high temperature Cvi maternal environment reduces stamen extension via reduced GA biosynthesis is unclear. However, it was reversed by applying GA, indicating that reduced GA levels are the cause of poor seed production from Bur in the Cvi environment. The reduced ovule number in Bur is also consistent with results demonstrating that overexpression of the pea GA catabolism gene *GA2ox2* in arabidopsis resulted in reduced ovule number (Singh *et al.*, 2010). Brassinosteroids are important components of plants stress responses (Hao *et al.*, 2013) and also implicated in ovule formation and GA signalling via DELLA proteins.

The reduction in ovule number seen in Bur grown in the higher temperature Cvi environment is consistent with the observation that heat stress reduces ovule number and increases abortion in arabidopsis (Whittle *et al.*, 2009). However, comparison of the two ecotypes here provided evidence of natural variation in this effect. There will have been less selection pressure for tolerance to heat stress in ecotypes such as Bur that have adapted to a cool climate than in those like Cvi adapted to warmer climates.

### Conclusions

The results presented show that high temperature in the maternal environment negatively affected the final seed yield of both Bur and Cvi ecotypes of arabidopsis, but the extent of this response differed, demonstrating natural variation. The high temperature reduction in seed yield in Bur was enhanced by failure of pollination due to reduced stamen extension relative to the pistil during the fertilization period. Higher maternal temperature also reduced seed dormancy and therefore enhanced germination.

Increases in mean global temperature and extremes of temperature are predicted for the future, and these predictions are significantly larger than those that have occurred to date. It is therefore important to understand how and whether plants can adapt their growth and developmental processes to the changing environment. Normally Bur, as a summer annual, flowers and sets seeds in late summer up to early autumn, and then overwinters as dormant seeds, which then germinate in spring. However, the results presented suggest that future higher summer temperatures, if above the optimum, could reduce seed yield and seeds could have more shallow dormancy. In an extreme scenario, Bur, instead of entering dormancy and overwintering as a seed, could potentially germinate soon after imbibition in early autumn, leading to a shift from spring to autumn emergence. However, under less extreme conditions, the seed would enter winter less dormant, and this would tend to offset the reduced dormancy-breaking effect of higher winter temperatures (low temperature relieves dormancy in Bur) tending to maintain the status quo. In contrast, reduced dormancy as a result of higher seed maturation temperatures is unlikely to alter germination timing of the winter annual Cvi. However, a potential consequence of lower dormancy at maturity and more effective dormancy relief from higher temperatures (high temperatures relieve dormancy in Cvi) may be that seeds germinate earlier in autumn or late summer, so plants enter winter as a larger rosette. The consequences of this are unknown, but may be beneficial.
